# Burden of care amongst caregivers who are first degree relatives of patients with schizophrenia

**DOI:** 10.11604/pamj.2017.28.284.11574

**Published:** 2017-11-30

**Authors:** Chinwe Frances Inogbo, Sunday Osasu Olotu, Bawo Onesirosan James, Emmanuel Okechukwu Nna

**Affiliations:** 1 Department of Clinical Services, Federal Neuropsychiatric Hospital Uselu, Benin City Edo State, Nigeria; 2 Safety Molecular Pathology Laboratory, Rangers Avenue Independence Layout Enugu, Enugu State, Nigeria

**Keywords:** First-degree relatives, caregiver burden, schizophrenia, burden of care, GHQ-12, Zarit burden interview

## Abstract

**Introduction:**

Caring for a mentally ill family member is a challenging task. Caregivers who are first-degree relatives (FDR) are at a higher risk of experiencing the negative consequences of caregiving. This study was aimed at determining burden of care and its correlates in caregivers who are first-degree relatives of patients with schizophrenia.

**Methods:**

A dyad of 255 patients and caregivers was recruited. A socio-demographic questionnaire was administered to both. The GHQ-12 was used to screen for psychiatric morbidity in the FDRs. Caregiver's burden was assessed with the Zarit Burden Interview. Patients' illness severity and level of functioning were assessed using the Brief Psychiatric Rating Scale and the Global Assessment of Functioning scales respectively.

**Results:**

The mean ± SD age of caregivers and patients were 45.1 ±12.3 and 36.7 ±13.4 years respectively. About 49% of caregivers experienced high burden of care. Older caregiver's age (r = 0.179; p < 0.004) and greater illness severity (r = 0.332; p < 0.0001) in the patient had weak to moderate positive correlation with burden of care. Caregiver's burden also increased with poorer functioning of the patient (r = -0.467 p < 0.0001). Independent predictors of caregiver burden were low level of education of the caregiver (OR 2.45; 95% CI 1.27-4.73), psychiatric morbidity in the caregiver (OR 6.74; 95% CI 2.51-18.15) and poor patient functioning (OR 2.81; 95% CI 1.27-6.18).

**Conclusion:**

Caregivers who are first-degree relatives of patients with schizophrenia experience varying degrees of burden of care during caregiving. Routine screening and early psychological intervention would help to ameliorate these negative consequences of caregiving.

## Introduction

Schizophrenia is a chronic psychiatric disorder characterized by dysfunction in one or more areas of functioning; interpersonal relations, work or education, or self-care [1]. It ranks among the 5^th^ and 6^th^ leading contributors to global disease burden among males and females respectively [2]. It runs a chronic course, which is characterized, by remission and relapses leading to deterioration in social functioning, occupational functioning [3] and a loss in productivity [1, 4]. The consequent economic impact of this disorder on both caregiver and care recipient is enormous. Direct and indirect costs arise from therapeutic interventions and loss of productivity respectively [1, 5]. These costs overlap with emotional and social burden resulting from care to sufferers [4,5]. In some healthcare systems, especially in low and middle income countries, direct costs are also borne by caregivers. In most cases, direct cost, which is largely out-of-pocket finance for healthcare, is borne mostly by relatives of individuals in their communities [6-9]. Caregivers often alter their household schedules and lifestyle to accommodate the special needs of ill relatives [10]. This could exacerbate the burden of care they experience [10, 11]. Burden of care has been categorized into objective and subjective burden [12]. Objective burden refers to outwardly quantifiable demands such as the financial cost of the illness, disruption of family routines, and patient's dependence on the family for both economic support and support with activities of daily living [13]. Subjective burden refers to the emotional response of the caregiver to the behavioural and social difficulties of the mentally ill [7]. Many patients with schizophrenia are mostly cared for in the communities by their relatives [14]. In Nigeria, these caregivers are mostly relatives who are likely to be mothers to the care recipients [15]. Care recipients are dependent majorly on their caregivers for their daily activities [14]. This causes restructuring of household schedules and disruption in family routines [10]. This contributes to increased burden of care. Caregivers of people with mental disorders are at risk of developing psychological distress due to increased caregiver burden [16, 17]. Caregivers who are biological relatives of persons with schizophrenia have higher risk of developing mental disorders due to their genetic predisposition [15]. This study aimed to determine the burden of care and its correlates in caregivers who are first-degree relatives of patients with schizophrenia.

## Methods

This cross-sectional study was conducted at the outpatient clinic of the Federal Neuro-Psychiatric Hospital (FNPH), Benin City, Edo State in Southern Nigeria. It is a tertiary government hospital that provides both in-patient and out-patient mental health services to the inhabitants of the state and its environs. The participants comprised of a ‘dyad’ of a first degree relative; who is the primary caregiver, and the patient with schizophrenia attending the out-patient clinic. A primary caregiver was defined as a first-degree relative and a non-professional, non-paid person who was mostly involved with the everyday care of the patient. He/she should also be very likely to respond to any request for special assistance at any time, if such a request was made by the patient [8, 18]. A first-degree relative could be a biological parent, a full sibling, or biological child of the patient. Caregivers who were aged 18 years and above, first-degree relatives and the primary caregivers of the patient were recruited. Caregivers who could not communicate in English or declined consent to participate in the study were excluded. Patients who were diagnosed as having schizophrenia by the Consultant Psychiatrist and diagnosis confirmed using the psychotic module of the Mini International Neuropsychiatric Interview (M.I.N.I.) were recruited for the study. Ethical approval was obtained from the Ethics and Research Committee of the hospital (FNPH, Benin City). Each dyad was recruited through systematic random sampling method over a period of 3 months. Both patients and caregivers were assessed using a socio-demographic questionnaire to ascertain variables like age, gender, level of education etc.

**Screening for psychiatric morbidity:** The General Health Questionnaire (GHQ)-12 is a 12 item, self-administered screening questionnaire, designed for use during consultations aimed at identifying people with a probable psychiatric morbidity. It is the most widely used screening instrument for common mental disorders and serves as a general measure of psychiatric well-being. Each item on the scale has four responses and it utilizes 2 scoring methods: bimodal scale (0-0-1-1) and a 4-point Likert-type scale (0-1-2-3). Total scores were calculated by summing up the total scores for each item using the bimodal method of scoring. It was used to screen for probable psychiatric morbidity in all the FDRs who are caregivers of people with schizophrenia. A GHQ -12 cut-off point of 4 was used to define probable psychiatric morbidity.

**Assessment for burden of care:** Caregivers were assessed for caregiver burden using the Zarit Burden Interview (ZBI). The ZBI is a 22-item questionnaire in which the caregivers report on their experiences of caregiving based on a 5-item Likert scale. Levels of burden are graded as: 0 to 20 points = little or no burden; 21 to 40 points = mild to moderate burden; 41 to 60 points = moderate to severe burden and 61 to 88 points = severe burden.

**Assessment of illness severity:** The Brief Psychiatric Rating Scale (BPRS) was used to assess the illness severity of the patients. It is an 18- item clinician rated instrument used to assess the level of severity of 18 symptom construct e.g. hallucinations, grandiosity, suspiciousness etc. in patients with psychosis. Total scores were obtained from the 7-point rating scale ranging from not present to extremely severe. Scores from each item were summed up to give a total score, which was the index of illness severity.

**Assessment of level of functioning:** The level of functioning for each patient was assessed using the Global Assessment of Functioning (GAF) scale. The GAF scale is a clinician rated assessment of the level of functioning of a patient. It is a numeric scale that rates subjectively: social, occupational and psychological functioning of adults. Data generated from the study was analyzed using the Statistical Package for Social Sciences (SPSS) version 22 and GraphPad Prism version 7. Summary statistics were done using frequencies and tables. The Zarit burden interview scores were dichotomized with a score of 24 and above implying high burden of care. Test of association between burden of care and categorical variables (gender, level of education, employment status etc.) were performed using the Pearson's Chi-square test. Mann Whitney U test was used to test for difference in numerical variables (age, duration of time spent in caregiving per day etc.) between high and low caregivers' burden. Binary logistic regression analysis was used to determine predictors of caregiver burden. Alpha level of significance was set a-priori as p < 0.05.

## Results

**Caregivers:** The mean age (SD) of the caregivers was 45.1 (12.3) years. The caregivers were mostly married (61.6%) females (65.5%) with secondary level of education (33.3%) and were employed (83.1%). One hundred and sixty-seven (65.5 %) caregivers earned more than N10, 000 ($50) monthly. A majority of the caregivers live with the patient (93.3%), have been caring for the patient for more than 48 months (52.9%) and are solely responsible for payment of their treatment (51%) (Table 1 and Table 2).

**Table 1:** Socio-demographics of caregivers and patients

Demographic Characteristics	Caregivers	Patients
**Age:** Mean (± SD) yrs.	45.1(12.03)	36.7 (13.4)
**Gender**		
Male	88	138
Female	167	117
**Religion**		
Christianity	250	249
Islam	3	4
African Traditional Religion	2	2
**Marital Status**		
Unmarried	98	228
Married	157	27
**Educational level**		
No formal education	21	20
Primary	69	63
Secondary	85	115
Post-secondary	29	27
Tertiary	51	30
**Employment status**		
Employed	212	55
Unemployed	43	200

**Table 2:** Other socio-demographic characteristics of caregivers

Variable	N	%	95% CI^++^
**Median Monthly Income (^+^$)**			
≤ Median ($50)	88	34.5	28.9- 40.5
> Median ($50)	167	65.5	59.5-71.1
**Monthly Expenditure ($)**			
≤ Median ($30)	130	51.0	44.9-57.1
> Median ($30)	125	49.0	42.9- 55.1
**Duration of care for patient since onset of illness**			
≤ 48 months	135	52.9	46.8-59.0
> 48 months	120	47.1	41.0-53.2
**Duration of time spent caring for the patient per day**			
≤ 12 hours	152	59.6	53.5-65.5
> 12 hours	103	40.4	34.6- 46.5
**Who pays for patient’s treatment**			
Caregiver alone	130	51.0	44.9- 57.1
Patient alone	8	3.1	1.5-6.2
Caregiver and patient	4	1.6	0.5-4.1
Other relatives	82	32.2	26.7-38.1
Caregiver and other relatives	31	12.2	8.7-16.8
**Relationship with the patient**			
Father	18	7.1	4.5-10.9
Mother	107	42.0	36.1- 48.1
Sibling	91	35.7	30.1-41.0
Child	39	15.3	11.4-20.3
**Are you currently living with the patient?**			
Yes	238	93.3	89.5-95.9
No	17	6.7	4.1-10.5

+ The current exchange rate is based on prevailing parallel market rate in Nigeria.

++ CI is Confidence Interval calculated by using modified Wald method (GraphPad Prism v7).

**Patients:** The mean age (SD) of the patients was 36.7 (13.4) years. There were mostly unmarried (89.4%) males (54.1%) with secondary level of education (45.1%) and were unemployed (78.4%). Median duration of illness was 72 months. The median BPRS and GAF scores were 30 and 60 respectively (Table 1).

**Burden of care:** One hundred and fifty-four (60.4%) caregivers experienced varying degrees of burden (mild-severe burden). One hundred and one (39.6%) caregivers reported little or no burden, ninety-six (37.6%) reported mild burden, forty-two (16.5%) had moderate burden and sixteen (6.3%) reported high burden. Caregiver's age, level of education, being a parent, being solely responsible for financing patient's treatment, poor perception of patient's improvement and psychiatric morbidity were significantly associated with greater burden of care (P value < 0.05). Caregiver's of younger patients who are unemployed with greater illness severity and poorer psychosocial functioning reported greater burden of care (Table 3). Caregiver burden increased with decreasing level of functioning in the patient (r = -0.467; p < 0.0001). Caregiver's age (r = 0.179; p < 0.004) and illness severity (r = 332; p < 0.0001) in the patient had weak to moderate positive correlation with burden of care (Table 4). Significant variables from the univariate analysis were entered into the Binary logistic regression model to determine independent predictors of burden of care among caregivers. Caregivers with lower level of education were about 3 times more likely to report greater burden of care compared to those with higher levels of education. Caregiver's with psychiatric morbidity were about 7 times more likely to report greater burden of care compared to those without psychiatric morbidity. Psychiatric morbidity also showed a positive trend with levels of burden of care (no or little burden, mild burden, moderate and severe burden) with Chi Square for trend value of 52.07, p value < 0.0001. Poorer functioning in the patients also predicted caregiver burden (Table 5).

**Table 3:** Comparison of socio-demographic and clinical characteristics between caregivers with high burden of care and those with low burden of care

Variables	P values
**Caregiver’s variables**	
Age	**0.02**
Gender	0.69
Level of education	**0.01**
Employment status	0.51
Relationship with patient (Parent)	**0.002**
Currently living with the patient	0.62
Who pays for patient’s treatment (Caregiver alone)	**0.001**
Total monthly income spent on care	0.07
Duration of time spent in caregiving per day	0.32
Caregiver’s perception of patient’s improvement	**0.001**
Psychiatric morbidity (GHQ – 12)	**0.0001**
**Patient’s variables**	
Age	**0.003**
Gender	1.00
Level of education	0.07
Employment status	**0.02**
BPRS scores	**0.0001**
GAF scores	**0.0001**

**Table 4:** Correlation amongst continuous and ordinal variables (patient and caregiver) and caregiver burden (ZBI scores)

Variable	Rho	P value
Caregiver’s age	0.179	**0.004**
Duration of care (months)	0.109	0.081
Duration of time spent in caregiving per day	0.105	0.093
Average monthly income	-0.007	0.907
Average monthly income spent on patient	0.043	0.495
Patient’s age	-0.201	**0.001**
Duration of illness	0.085	0.174
GAF	-0.467	**0.0001**
BPRS	0.332	**0.0001**

**Table 5:** Predictors of burden of care among caregivers

Variables	Exp (β)	SE	Wald	Significance
Caregiver’s level of education	2.452	0.336	7.139	0.008
Patient’s GAF scores	2.805	0.403	6.561	0.010
Caregiver’s Psychiatric morbidity	6.742	0.505	14.274	0.0001

## Discussion

We identified varying levels of caregiver burden among caregivers who are first-degree relatives. Nearly half of the caregivers reported significant burden (mild to severe burden). Although this agreed with previous findings of significant burden among caregivers [6-8, 19, 20], the rates were lower for our study. An explanation may be that first-degree relatives (FDRs) who are caregivers of the patients were sampled. These FDRs may view their caregiving role as an obligation hence, their reluctance to report negative experiences of caregiving. Our study found that caregiver's low level of education (i.e. secondary education and below) was a significant predictor of caregiver burden. This finding agrees with similar reports from previous studies [9, 19, 20]. Low educational attainment is associated with low income and socioeconomic status. Caregivers with low educational status may be socially and economically disadvantaged. They may lack the financial capacity to meet up with the demanding needs of caregiving. In addition, lower educational attainment may negatively affect caregiver's knowledge and understanding of the illness. Poor psychosocial functioning of the patient was also an independent predictor of caregiver's burden. We found that the poorer the patient's functioning the greater the level of burden. Patient's poor functioning may imply greater illness severity. Similar to other studies [13, 21,22], caregivers of patients with greater illness severity reported greater burden of care. Greater illness severity implies presence of florid psychotic symptoms, poor insight and non-adherence to medication, frequent hospital admissions, and increased cost of care. Care recipients become more dependent on the caregivers causing a disruption in their family routines. These factors worsen both objective and subjective burden.

High burden of care was significantly associated with occurrence of psychiatric morbidity in caregivers. Generally, the association between psychiatric morbidity and caregiver burden have been previously reported [6, 7, 18, 23-25]. The caregiving experience is considered a chronic stressor which impacts negatively on the mental health of caregivers [26]. Chronic stress could cause physiological changes which ultimately lead to psychiatric morbidity [26]. On the other hand, caregivers who are FDRs, due to shared genetic makeup with the care recipient have greater vulnerability to develop psychiatric morbidity [27]. Caregivers with pre-existing psychiatric morbidity may experience greater burden or rate their perception of burden higher.

## Conclusion

The relevance of this study is far reaching. Caregivers, who are first-degree relatives of patients with schizophrenia, have significant burden of care, which are associated with presence of psychiatric morbidity in the caregivers themselves, the patient's poor functioning status and the educational status of the caregiver. Adequate screening of caregivers and early psychological interventions might be necessary as a means of supporting first-degree relatives who are involved in caregiving. Limitations: the cross-sectional design of this study did not allow for the determination of a temporal relationship between psychiatric morbidity and caregiver burden. The effects of stigma, knowledge of illness and poor medication adherence on caregiver burden were not assessed.

### What is known about this topic

- Caregivers of people with schizophrenia experience negative consequences of caregiving;
- Caregiver burden is highest among relatives of people with schizophrenia.

### What this study adds

- Caregivers who are first degree relatives of people with schizophrenia experience varying degrees of burden;
- Psychiatric morbidity and poor educational status of caregivers predicts caregiver burden;
- Caring for a relative with poorer psychosocial functioning also was a predictor of caregiver burden.

## Competing interests

The authors declare no competing interests.
